# Association Between Depressive Symptoms and Self-Reported Ongoing Medication Use Among Pharmacy Students: A Cross-Sectional Study

**DOI:** 10.3390/healthcare14131851

**Published:** 2026-06-25

**Authors:** Reynaldo Arellano-Cervantes, Raymundo Escutia-Gutiérrez, Nancy Evelyn Navarro-Ruiz, Erika Fabiola López-Villalobos, María Luisa Muñoz-Almaguer, Karime Lilian Franco-Pérez, Diana Esperanza Arévalo-Simental, Aline Priscilla Santiago-García, J Ahuixotl Gutiérrez-Aceves, Delia Flores-Avila, Tammy Marah Estrella Vergara-de la Torre, Santiago José Guevara-Martínez, Cesar Ricardo Cortéz-Álvarez, Felipe Alexis Avalos-Salgado

**Affiliations:** 1 Departamento de Ciencias del Movimiento Humano, Centro Universitario de Ciencias de la Salud (CUCS), Universidad de Guadalajara, Guadalajara 44340, Mexico; reynaldo.arellano7122@academicos.udg.mx; 2 Departamento de Farmacobiología, Centro Universitario de Ciencias Exactas e Ingenierías (CUCEI), Universidad de Guadalajara, Guadalajara 44430, Mexico; raymundo.escutia@academicos.udg.mx (R.E.-G.); maria.malmaguer@academicos.udg.mx (M.L.M.-A.); santiago.guevara@academicos.udg.mx (S.J.G.-M.); cesar.cortez@academicos.udg.mx (C.R.C.-Á.); 3 Departamento de Vida Saludable y Promoción de la Salud, Centro Universitario de Tlajomulco, Universidad de Guadalajara, Tlajomulco 45641, Mexico; nancye.navarro@academicos.udg.mx; 4 Laboratorio de Análisis Clínicos e Investigación Traslacional, Centro Universitario de Ciencias Exactas e Ingenierías (CUCEI), Universidad de Guadalajara, Guadalajara 44430, Mexico; erikafabiola.lopez@academicos.udg.mx; 5 Independent Researcher, Guadalajara 45030, Mexico; karimefrancopz@gmail.com (K.L.F.-P.); priscillasga1703@gmail.com (A.P.S.-G.); 6 Jefatura de Servicio de Retina Médica y Quirúrgica, Hospital Civil de Guadalajara “Fray Antonio Alcalde”, Guadalajara 45019, Mexico; diana.arevalo@academicos.udg.mx; 7 División de Educación e Investigación en Salud, Hospital de Especialidades, Centro Médico Nacional de Occidente, Instituto Mexicano del Seguro Social, Guadalajara 44329, Mexico; ahuixotl@gmail.com; 8 Clínica Medicina Familiar No. 2, Santa Teresita Instituto de Seguridad y Servicios Sociales de los Trabajadores del Estado (ISSSTE), Guadalajara 44600, Mexico; delia.flores@issste.gob.mx; 9 Departamento de Biología Molecular y Genómica, Centro Universitario de Ciencias de la Salud (CUCS), Universidad de Guadalajara, Guadalajara 44340, Mexico; tammymarahestrella@gmail.com

**Keywords:** depression, mental health, pharmacy students, medication usage, well-being, depressive symptoms, Patient health questionnaire (PHQ-9)

## Abstract

**Background/Objectives:** Depression is a mood disorder characterized by persistent sadness and loss of interest. Pharmacy students exhibit a relatively high prevalence of depressive symptoms, which may negatively impact quality of life, academic performance, and, in severe cases, lead to suicidal ideation. Given the increasing trend of medication use among young adults, we hypothesized that this behavior may be associated with depressive symptoms, potentially reflecting attempts to manage concurrent physical symptoms or reduced psychological well-being. Therefore, the objective of this study was to assess the association between depressive symptoms and medication use among pharmacy students. **Methods:** A cross-sectional study was conducted among students enrolled in pharmacy-related programs from University Center for Exact Sciences and Engineering (CUCEI), University of Guadalajara. Participants completed a personalized questionnaire assessing sociodemographic variables, medication use, comorbid conditions, and depressive symptoms using the Patient Health Questionnaire-9. Descriptive statistics were used to summarize participant characteristics. Categorical variables were reported as frequencies and percentages and compared using the chi-square test. Continuous variables were summarized as means and standard deviations and compared using Student t-test. To evaluate factors associated with moderate-to-severe depressive symptoms, logistic regression and multivariable linear regression analyses were performed. **Results:** A total of 365 students completed our personalized questionnaire; nearly half of the sample (47.3%) presented moderate-to-severe depressive symptoms. Multivariable analyses showed that insufficient sleep, academic stress, psychological support, and the number of medications used simultaneously were significantly associated with depressive symptoms. Logistic regression identified being in a relationship and receiving psychological support for at least one year as protective factors, while employment, insufficient sleep, academic stress, and a greater number of concomitant medications were associated with increased odds of moderate-to-severe depressive symptoms. **Conclusions:** A modest association was observed between self-reported medication use and moderate-to-severe depressive symptoms among pharmacy students. These findings suggest that medication use patterns may warrant further investigation as a potential marker of depressive symptoms in pharmacy students. Future longitudinal studies are needed to clarify the nature and direction of this association and to determine whether medication use could contribute to the identification of students at increased risk of depression.

## 1. Introduction

Depression is a mood disorder that causes a persistent feeling of sadness and loss of interest, characterized by emptiness, or irritable mood, accompanied by somatic and cognitive changes that significantly affect the individual’s capacity to function [1]. College students experience higher levels of depression when compared to the general population [2]. Depression tends to be undiagnosed because its signs and symptoms tend to be confused or misunderstood, or within certain cultural contexts, emotional experiences may be downplayed, and individuals may be expected to suppress or ignore their emotions; therefore, consequences could only be observed on individuals with severe episodes [3,4]. Depressive symptoms among students have been associated with impaired academic and social functioning, reduced quality of life, substance misuse, absenteeism, and higher dropout risk, while severe symptomatology may increase the likelihood of suicidal ideation [5,6,7,8]. Risk factors associated with depression, and therefore increasing depressive symptoms, are: academic stress, sleep disruption, social pressure that is often amplified by social media use, financial stress, and uncertainty about the future alongside life-stage transitions such as graduating and entering the workforce [9,10,11,12,13]. Pharmacy students, like those enrolled in any other health-related professional programs, are required to acquire extensive knowledge across multiple disciplines, such as biology, pharmacology, quality assurance, and genetics [14]. In addition to their academic coursework, they must gradually integrate into professional practice through internships and social service placements [15,16]. These simultaneous academic and professional demands may represent significant sources of stress and psychological burden. In this context, the Patient Health Questionnaire-9 is an important screening tool because it facilitates the early identification and quantification of depressive symptoms, enabling timely evaluation and intervention before symptom progression becomes more severe [17].

Given the increasing prevalence of depressive symptoms among university students, it is also important to evaluate health-related behaviors potentially associated with mental health status, including medication use [18,19]. Nowadays, an increasing trend in medication use has been observed among young people, particularly for the management of physical symptoms such as headaches and pain, regardless of whether these symptoms are associated with underlying pathological conditions or transient discomfort [20,21,22]. The widespread availability of over-the-counter medications and the common practice of self-medication, the practice of treating one’s own physical or psychological symptoms using medications, substances, or home remedies without supervision or diagnosis of a healthcare professional [2,3], may further contribute to increased medication exposure among young adults [23,24]. Likewise, a phenomenon of overprescription has been observed within healthcare systems, facilitating easier access to medications for patients [25,26]. The most used drugs identified are analgesics, anti-inflammatories, stomach protectors or antiacids, and antihistamines [27].

The increase in medication use is important to assess because pharmacological agents may contribute to depressive symptoms either as adverse effects or through mechanisms that influence neurotransmitter activity, appetite, fatigue, or sedation [28]. Consequently, students exposed to a greater number of active pharmaceutical ingredients may present a higher probability of experiencing adverse effects affecting the central nervous system, potentially contributing to the onset or worsening of depressive symptoms. Although depression is recognized as a multifactorial condition influenced by biological, psychological, social, and environmental factors, medication use may represent an additional factor associated with depressive symptomatology or a marker of underlying conditions related to mental health. The relationship may also be bidirectional, where potential clinical confounders, including underlying health conditions and symptom burden, may contribute to both increased medication use and depressive symptoms.

Despite the increasing use of medications among young adults and the potential neuropsychiatric effects of several pharmacological agents, limited evidence has examined whether medication use patterns, particularly the concurrent use of multiple medications, are associated with depressive symptoms among pharmacy students. Understanding this relationship may help determine whether medication use could serve as a potential marker of vulnerability to depressive symptoms in this population. Therefore, the objective of this study was to evaluate the association between self-reported use of medications taken for at least two weeks and depressive symptomatology, as measured by the PHQ-9, among pharmacy students.

## 2. Materials and Methods

### 2.1. Study Design and Clinical Setting

Study design: Cross-sectional study. We included pharmacy students from Universidad de Guadalajara in Mexico. The study was performed from July 2025 to December 2025.

### 2.2. Inclusion and Exclusion Criteria

Participants were students currently enrolled in pharmacy-related programs. These students were ≥18 years old. Students were excluded if they did not complete all questionnaires or wished to withdraw from the study. Students with any condition or disease leading to disability to answer questionnaires were also excluded.

### 2.3. Variables and Measurement

Demographic, clinical and pharmacological data were ascertained by using a structured questionnaire. Information collected was classified as:

- Sociodemographic variables: Gender, age, social media usage, exercise and resting habits.
- Clinical characteristics: Students were asked to report if they had any disease (i.e., asthma, diabetes, etc.), and if they presented any physical symptoms, which were defined as the self-reported presence of persistent or recurrent bodily symptoms, including headache, gastrointestinal symptoms, musculoskeletal pain, respiratory symptoms, skin conditions, vision problems, hormonal disorders, or recurrent infections. Depression symptoms were explored using the Patient Health Questionnaire-9 (PHQ-9) test [17,29]. The (PHQ-9) is a self-administered questionnaire consisting of nine items that assess the frequency of depressive symptoms over the previous two weeks. Each item is scored from 0 to 3, resulting in a total score ranging from 0 to 27. Scores are commonly categorized as none (0–4), mild (5–9), moderate (10–14), moderately severe (15–19), and severe depression (20–27). Alongside the structured questionnaire, each student was given a printed copy of the PHQ-9 to answer, subsequently, we calculated the total points of each student using the statistical program. Based on these results, students were dichotomized into 2 groups, group (a) Students without depression or with mild symptoms (0 to 9); and group (b) Students with moderate to severe depression symptoms (10 to 27).
- Pharmacological data: Students were asked if they used any type of medication for a period equal or longer than 2 weeks; this temporal restriction was placed in order to identify ongoing or sustained treatments and reduce the influence of temporary medication use related to acute conditions. Drugs were classified based on their therapeutic class and also were categorized according to Mexican legislation [30] as over-the-counter (OTC) or prescription-only medications. No prescriptions or medical records were requested to verify the reported use of prescription medications. Psychotropic medication was defined as the use of antidepressants, anxiolytics, or antipsychotic medications.

### 2.4. Study Development and Study Size

A convenience sampling strategy was employed. Pharmacy students were approached by two trained researchers during their free time and invited to participate in the study. Students who agreed to participate provided written informed consent before data collection. A total of 410 students participated, while 45 students declined participation. Information regarding the total number of students enrolled in the program during the study period was not available; therefore, a population-based participation rate could not be calculated. The minimum sample size was calculated assuming a depression prevalence of 40%, a Zα value of 1.96 (corresponding to a 95% confidence level), and an expected margin of error of 5%, resulting in a required sample size of 368 students.

### 2.5. Biases

Potential sources of bias include variability in the timing and context of data collection, as surveys were administered throughout the school day and information regarding recent examinations, academic activities, meals, or academic workload was not collected. Consequently, differences in short-term stress levels and course burden may have influenced participants’ responses. Selection bias is also possible because participation was voluntary, and students with different mental health profiles may have differed in their willingness or availability to participate. Additionally, non-response bias may have occurred if students who declined participation differed systematically from respondents. Finally, depressive symptoms were assessed through self-report questionnaires, introducing the possibility of reporting and social desirability biases.

### 2.6. Statistical Analysis

Two groups were conformed based upon the score of PHQ-9. Group A was defined as students without depression or with mild symptoms, while group B was defined as students with moderate to severe depression symptoms. Comparison between qualitative variables were performed by using chi-square test and quantitative variables were compared by using Student t-test. A multivariable general linear model was conducted to assess the effect of risk variables associated with the PHQ-9 score, variables with a *p*-value < 0.20 in the bivariate analysis, as well as those considered relevant based on the existing literature, were included in the multivariable model. The significance level was set at *p* ≤ 0.05. The analysis was performed using the statistical software SPPS Statistics Version 24.

## 3. Results

A total of 365 pharmacy students consented to participate in the study, while 45 students were excluded for the following reasons: 36 did not complete the questionnaire, and 9 declined to participate. From these, a total of 192 (52.7%) pharmacy students reported none or mild depressive symptoms, accounting for group A; whereas, 173 (47.3%) pharmacy students reported moderate to severe depressive symptoms, accounting for group B. Table 1 compares the sociodemographic characteristics of the students in group A and students in group B included in this study. Female gender was more frequent among students with moderate to severe symptoms (69.9% vs. 57.8%, *p* = 0.016). Age and relationship status did not differ significantly between groups. Students with moderate to severe depressive symptoms reported higher employment (43.9% vs. 33.9%, *p* = 0.048), and comorbidities (14.5% vs. 6.8%, *p* = 0.016). They also had a higher prevalence and number of physical symptoms (75.1% vs. 52.1% and 2 ± 1 vs. 0 ± 1, respectively; *p* ≤ 0.001 for both). Additionally, this group reported greater Instagram use (86.1% vs. 77.6%, *p* = 0.024), sleep deprivation (34.7% vs. 66.7% reporting being well rested, *p* ≤ 0.001), higher school stress (88.3% vs. 72.0%, *p* ≤ 0.001), more family problems (62.0% vs. 35.7%, *p* ≤ 0.001), and greater use of psychological support for ≥1 year (17.9% vs. 7.3%, *p* = 0.002). No differences were observed in total hours of social media use, WhatsApp use, exercise, or pet ownership.

Considering that the use of psychotropic medications (antidepressants, antipsychotics, or anxiolytics) could itself represent a source of bias in the analysis, a second comparison was performed excluding all students using any of these medications (n = 21 excluded). Table 2 presents this analysis, in which the factors previously associated with moderate to severe depressive symptoms remained statistically significant.

Table 3 compares pharmacological treatments among groups. Students with moderate to severe depression symptoms showed significant difference in the usage of medication (32.4% vs 18.8%, *p* = 0.003), and the usage of antidepressants, antidiabetic medications and hormonal therapy also showed to be more prevalent in this group (*p* ≤ 0.050). Antidepressants accounted for the most used medication in the group with moderate to severe depression symptoms (8.1%), from which the most common class was selective serotonin reuptake inhibitors (SSRIs) (5.1%), followed by serotonin–norepinephrine reuptake inhibitors (SNRIs) (2.3%) and tricyclic antidepressants (0.6%). The second most frequently reported medications were hormonal medications (6.9%), followed by nonsteroidal anti-inflammatory drugs (NSAIDs) and analgesics (6.4%). It must also be noted that all dermatological medications reported corresponded to the use of retinoids for skincare. Finally, we compared the mean number of medications used by each group, observing a statistical difference in students with moderate to severe depression symptoms (1.0 ± 1.0 vs. 0.0 ± 1.0, *p* ≤ 0.001).

Table 4 describes and compares the prevalence of self-reported physical symptoms between groups. Students with moderate to severe depressive symptoms reported a higher prevalence of several health conditions compared with those without depression or with mild symptoms. A history of depression was significantly more common in this group (23.7% vs. 5.2%, *p* < 0.001). They also reported higher rates of vision problems (26.6% vs. 14.6%, *p* = 0.004), recurrent headaches (45.1% vs. 22.4%, *p* < 0.001), gastrointestinal symptoms (28.9% vs. 13.5%, *p* < 0.001), and musculoskeletal symptoms (26.6% vs. 13.5%, *p* = 0.002). Respiratory symptoms (7.5% vs. 2.1%, *p* = 0.014), skin conditions (16.2% vs. 6.8%, *p* = 0.004), and hormonal disorders (22.5% vs. 5.7%, *p* < 0.001) were also more frequently reported among students with moderate to severe depressive symptoms. No significant difference was observed in the prevalence of recurrent infections between groups (4.6% vs. 2.6%, *p* = 0.2).

A multivariable general linear model was conducted to evaluate factors associated with PHQ-9 depression scores. The overall model was statistically significant (*F*(13,364), *p* < 0.001) and explained 27.7% of the variance in PHQ-9 scores (R^2^ = 0.277; adjusted R^2^ = 0.257). Several variables were found to be significantly associated with higher PHQ-9 scores. Not feeling well rested showed the strongest association (*p* < 0.001; partial η^2^ = 0.128), followed by school stress (*p* < 0.001; partial η^2^ = 0.051). Receiving psychological help for ≥1 year (*p* = 0.009; partial η^2^ = 0.020) and the number of drugs taken (*p* = 0.005; partial η^2^ = 0.012) were also significantly associated with PHQ-9 scores (Table 5). Gender, relationship status, employment, social media use (hours), reported at least one comorbidity and age were not significantly associated with PHQ-9 scores in the adjusted model.

Table 6 shows the results of multiple logistic regression analyses; in the model we included as a dependent variable: presenting moderate to severe depressive symptoms by PHQ-9. Covariables (potential confounders) tested in the unadjusted model (enter method) were: gender, age, if they were in a relationship, if they were currently working, comorbidity, if they had insufficient sleep, level of school stress, if they sought psychological help (≥1 year), hours of social media usage and number of drugs. The risk model showed significant relations between presenting moderate to severe depressive symptoms with students in a relationship (OR = 0.57, 95% CI = 0.35–0.91, *p* = 0.021), currently working (OR = 1.69, 95% CI = 1.03–2.78, *p* = 0.037), with insufficient sleep (OR = 3.12, 95% CI = 1.96–4.97, *p* ≤ 0.001) and feeling school stress (OR = 2.88, 95% CI = 1.51–5.49, *p* = 0.001). After adjusting by the stepwise method, these potential confounders remained showing a statistical relation with the dependent variable: students in a relationship (aOR = 0.61, 95% CI = 0.38–0.98, *p* = 0.042), currently working (aOR = 1.73, 95% CI = 1.07–2.77, *p* = 0.023) insufficient sleep (aOR = 3.37, 95% CI = 2.13–5.31, *p* ≤ 0.001) and school stress (aOR = 2.80, 95% CI = 1.50–5.22, *p* = 0.001). Additionally, the adjusted model presented two other variables associated with depressive symptoms: having psychological help for at least 1 year (aOR = 0.45, 95% CI = 0.21–0.96, *p* = 0.039) and the number of drugs taken simultaneously (aOR = 1.42, 95% CI = 1.05–1.92, *p* = 0.022).

## 4. Discussion

This study identified that almost half of the students (47.3%) presented moderate to severe depressive symptoms. The main risk factors associated with depressive symptoms were currently working, insufficient sleep, school stress and the number of drugs taken simultaneously, while being in a relationship and having psychological help acted as protective factors. The prevalence of medication usage was almost double in the group with severe depressive symptoms. The most used drugs identified were antidepressants, NSAIDS and painkillers, alongside hormonal medications.

### 4.1. Depression Prevalence and Factors Associated

In the present study, we observed that 47.3% of the students presented moderate to severe depressive symptoms, although these levels could be underrepresented based on how PHQ-9 was administered in our study. A written questionnaire was administered to each student; therefore, the perceived level of anonymity may have been reduced and concerns about social judgment may have increased, potentially leading to lower disclosure of depressive symptoms. A meta-analysis performed by Li W et al. reported a pooled depression prevalence of 33.6%, being the highest level in the Africa region of 40.1% [31]. Another study performed in Mexican medical students by Melo-Carrillo A et al. reported that 36.2% of the students reported depressive symptoms using the Beck depression inventory test [32]. A study with a similar prevalence was performed by Luo M et al. in Chinese college students also using PHQ-9 as screening tool, where they found levels up to 48.9% of depression [33]. Elevated prevalence of depressive symptoms has been observed in student populations across regions including Africa, China, and Mexico, highlighting substantial heterogeneity and suggesting that depressive symptoms may reflect context-dependent responses to environmental, academic, and sociocultural stressors. In the Mexican context, rising levels of violence, substance use, poverty, and perceived insecurity may constitute chronic environmental stressors that contribute to the elevated prevalence of depressive symptoms [34,35,36].

In our study, we observed well-established factors associated with depression, such as being female, working while studying, disease and physical factors, Instagram use, sleep-deprivation, school stress and family problems. Epidemiology studies have shown that depression is more common among women, although it has not been analyzed if this is due to physiological differences or cultural changes, where women tend to seek more help therefore increasing self-report numbers [37,38] while men tend to report fewer depressive symptoms even when experiencing similar or stronger distress, because men are expected to be strong by gender norms [39]. Job strains have been shown to precipitate clinical depression among employees [40]; therefore, it is expected that students that must attend school and are also required to work present higher depressive symptoms. Depression has been associated with developing physical illness [40], the burden of physical symptoms, including chronic pain, fatigue, and sleep disturbances, can impair daily functioning and reduce overall well-being. As these symptoms interfere with academic, social, and personal activities, individuals may experience a loss of autonomy and effectiveness, leading to frustration, decreased self-efficacy, and a diminished sense of control, which may contribute to the development of depressive symptoms. Reports of abuse and misuse of social media has been identified due to the perceived need of digitalization and the desire of social attention [41]. In our study, we identified that the two most used social media platforms by our students were Instagram and WhatsApp, although only the first one was associated with depressive symptoms, which could be explained due to differences in platform usage and content availability, where Instagram makes viewing easier. Nimbalkar S et al. also found that Instagram was correlated to depression in adolescents and young adults [42]. Sleep deprivation is strongly associated with depressive symptoms through interconnected biological and behavioral pathways. Disruptions in sleep can alter circadian regulation and neurotransmitter balance, impairing emotional processing and stress response [10,43]. Faisal R and Kimani J observed a strong association between inadequate and depressive symptoms through a survey performed in adolescents [44]. We observed in our sample that sleeping was the main factor in our linear model for higher scores in PHQ-9. The second most influential factor was stress related to academic performance. School-related stress is associated with depressive symptoms through sustained academic pressure, high performance expectations, and perceived lack of control. As stress persists, students may experience fatigue, disengagement, and feelings of inadequacy, which can contribute to the development and maintenance of depressive symptoms [45,46].

Students with moderate-to-severe depressive symptoms reported a significantly higher frequency of multiple physical symptoms and medical conditions, including recurrent headaches, gastrointestinal complaints, musculoskeletal symptoms, skin conditions, hormonal disorders, and respiratory symptoms. These findings are consistent with the established somatic manifestations of depression, in which psychological distress may coexist with, or exacerbate, physical symptom perception [47,48]. Furthermore, chronic stress and depressive symptomatology have been associated with alterations in sleep, pain perception, gastrointestinal function, endocrine regulation, and inflammatory pathways, potentially contributing to the higher burden of physical complaints observed in this group. Physical symptoms and chronic conditions may contribute to depressive symptoms, while depression itself may also increase symptom awareness, healthcare utilization, and self-reported morbidity. This increased burden of physical symptoms may partially explain the higher prevalence of medication use observed among students with moderate-to-severe depressive symptoms. Nevertheless, due to the cross-sectional nature of the study, causality cannot be established; also, it must be noted that neither physical symptoms nor previous diagnoses of depression were included in any model although both showed significance in bivariate analysis. Previous self-reported cases of depression were not considered as there were no reliable means of verifying self-reported diagnoses. Given the young age of the participants and the potential for recall bias, some students may have misremembered or misinterpreted past emotional experiences or encounters with mental health professionals. Since physical symptoms were not contemplated for not using a validated instrument or standardized scale to assess or confirm these symptoms, and given the inherently subjective nature of such reports, we considered this variable insufficiently reliable for inclusion in the analysis. Physical symptoms may act as common underlying causes of both medication use and depressive symptomatology, potentially confounding the observed association. The inclusion of validated measures for this variable, together with access to clinical records when feasible, may help reduce residual confounding and clarify the nature of the relationships identified in this study.

Protective factors observed in our study were being in a relationship and having psychological help. Close interpersonal relationships may provide emotional and social support, which can help individuals cope with academic, personal, and financial stressors commonly experienced during university life. Romantic relationships may also contribute to a greater sense of belonging and reduce feelings of loneliness. Pietromonaco et al. observed that greater partner responsiveness was associated with lower levels of depressive symptoms, potentially through enhanced emotional support and interpersonal functioning, although their findings were derived from a population of married couples [49]. Li X et al. analyzed a sample of Chinese high school students, observing too that having interpersonal trust reduced depression-related symptoms [50]. Receiving psychological support was also associated with lower odds of moderate-to-severe depressive symptoms. Psychological interventions may help individuals develop coping strategies, improve emotional regulation, and better manage stressors associated with academic and personal life. Yamamoto A et al. examined the efficacy of interpersonal counseling in undergraduate Japanese students, observing that learning coping mechanisms can reduce depressive symptoms [51].

### 4.2. Medication Usage

In our analysis we observed that students with more severe symptoms used more drugs in comparison to those with milder symptoms. The most commonly reported medications in this group were antidepressants. The persistence of severe depressive symptoms among students using antidepressants may reflect the fact that participants were assessed while actively undergoing treatment, before achieving full symptom remission. Other possible explanations include partial treatment response, inadequate adherence or ongoing psychosocial stressors. However, due to the cross-sectional design, methodological limitations, and lack of longitudinal data, it is not possible to evaluate these hypotheses or determine the temporal relationship between antidepressant use and depressive symptoms. Almarghalanu D et al. also explored the prevalence of antidepressants in medical students in Saudi Arabia, finding a slightly higher level of usage (23.8%), linking their usage to the lack of sleep, lack of physical activity, and family history of psychiatric disorders [52]. Fasanella N et al. also explored the prevalence of antidepressants in Brazilian medical students, finding also higher levels than ours, approximately 25.0%, noting that they were the most prescribed drug in their sample, mostly used to treat anxious or depressive symptoms, followed by insomnia [53]. Another study performed on Brazilian students, although they were enrolled in a pharmacy course, by Amaral C et al. observed a similar prevalence of antidepressant usage of 13.1%, being associated with economic matters, spirituality, and psychological care [18]. Other relevant psychotropic medications identified in our sample included antipsychotics and anxiolytics, although due to the small number of students using antipsychotics or anxiolytics, these medications were grouped together with antidepressants under the broader category of psychotropic medications; however, the broader category did not identify additional medication users reducing prevalence usage, resulting in insufficient statistical power to detect significant associations. Lower percentages of use could be explained by the relatively young and non-clinical nature of the population, where severe psychiatric disorders requiring these medications are expected to be less prevalent compared with depressive or stress-related symptoms.

Students using hormonal medications showed higher depressive symptom scores; although this study did not explore causality, hormonal imbalance has previously been associated with depressive symptoms and mood disorders [54,55]. Therefore, the observed association between hormonal medication use and depressive symptoms should be interpreted cautiously. Future studies should further distinguish between hormonal drugs prescribed for the treatment of medical conditions and those used for contraceptive purposes in order to better understand their potential relationship with depression [56,57].

Painkillers and NSAIDS were among the most frequently used therapeutic agents in both groups. Although their use was not statistically significantly associated with depressive symptoms, their high prevalence represents an interesting finding. Its use could be explained as a response to the high prevalence of headaches and musculoskeletal symptoms reported by the students, alongside the stress levels. Faqihi A and Sayed SF explored the self-medication practice focusing on NSAIDs in nursing undergraduates in Saudi Arabia, finding that almost half of the sample used them mostly to treat acute episodes of pain or discomfort [58]. Rajab M et al. observed that medical students started to use painkillers during exam periods firstly for pain management, but also for relaxation and to improve sleep and stay alert, thereby using them as a coping mechanism [59]. A similar pattern may be present in our sample, potentially driven by high levels of academic stress in conjunction with reported physical symptoms.

Regarding dermatological medications, all students in this category reported using isotretinoin. An association was observed between isotretinoin use and depressive symptoms; however, the interpretation of this finding is limited by the lack of information regarding treatment indications, disease severity, treatment duration, and other clinical characteristics. Consequently, it is not possible to determine whether the observed association reflects factors related to the medication itself, the underlying dermatological condition, or other unmeasured variables. Unlike other therapeutic groups, antihistamine use did not differ substantially according to depressive symptoms. Finally, the use of antidiabetic medications was observed in a small proportion of students; however, a statistically significant difference was identified between groups, with a prevalence of 0.5% in the low PHQ-9 group and 3.5% in the high PHQ-9 group.

As described by Celano C et al., pharmacological agents could lead to depressive symptoms either as an adverse event or a side effect; this could be explained by a mechanism of action which alters neurotransmitter levels, or indirectly by reducing appetite, causing fatigue or sedation [28]. Consequently, exposure to a greater number of active pharmaceutical ingredients may be associated with an increased likelihood of experiencing adverse effects involving the central nervous system. In the present study, students with moderate-to-severe depressive symptoms reported using a higher mean number of medications than those with lower symptom levels. However, this finding should be interpreted with caution, as the observed difference corresponded to approximately one additional active pharmaceutical ingredient. Therefore, further studies are needed to determine whether this association is clinically meaningful and to clarify the mechanisms underlying this relationship.

### 4.3. Strengths and Limitations

This study has several strengths. It represents an original contribution to the literature by exploring medication use as a potential indicator of depressive symptoms in a pharmacy student population. The relatively large sample size enhances the robustness and generalizability of the findings. Additionally, the use of validated assessment tools, such as the PHQ-9, strengthens the reliability of the measurements. The analytical approach, including the application of a multivariable linear model and logistic regression model, allowed for a more comprehensive evaluation of associations while accounting for potential confounding variables. Furthermore, the inclusion of behavioral habits provides a more integrative perspective, capturing relevant lifestyle factors that may influence both medication use and depressive symptoms.

However, it also includes several limitations. First, the cross-sectional design precludes the assessment of temporal and potentially bidirectional relationships between medication use and depressive symptoms, limiting the ability to determine the direction of the observed association. Second, medication use was assessed solely through self-report, and no prescription records were reviewed or verified. Consequently, information regarding prescribing practices, clinical indications, and adherence to treatment was unavailable, limiting the interpretation of the observed associations. Another limitation of this study is the lack of information regarding dietary patterns and eating habits, factors that may be associated with emotional regulation and mental health. Additionally, no access to participants’ medical records was available, preventing the verification of self-reported medication use and the assessment of relevant clinical information, such as underlying medical conditions, treatment indications, and psychiatric history. Consequently, the potential influence of these factors on the observed associations could not be evaluated. The reliance on self-reported data introduces the possibility of reporting bias, including recall bias and under- or overestimation of both medication use and depressive symptoms. Given the exploratory nature of this cross-sectional study and the number of univariate comparisons performed, the possibility of type I error inflation cannot be excluded. Therefore, statistically significant findings should be interpreted with caution and confirmed in future studies. Regarding insufficient sample size, even though the final sample size was three participants below the calculated minimum sample size, this difference was minimal (<1%) and is unlikely to have substantially affected the statistical power or precision of the study findings. An additional methodological limitation is the use of stepwise logistic regression for variable selection. Although this approach facilitates identifying variables independently associated with moderate-to-severe depressive symptoms and to reduce model complexity, it may lead to unstable variable selection, overfitting, and biased coefficient estimates, particularly when predictors are correlated. Therefore, the identified associations should be interpreted cautiously and confirmed in future studies using alternative modeling strategies and independent datasets.

### 4.4. Generalizability

Our sample was drawn from one of the largest public universities in Mexico [60,61], whose student population may partially reflect the characteristics of middle- to lower-income students attending public higher education institutions nationwide. However, caution should be exercised when generalizing these findings, particularly to students from private universities or institutions with different sociodemographic characteristics. Therefore, the present study should be considered exploratory and hypothesis-generating, aiming to evaluate a potential association between medication use and depressive symptoms among university students. Future research should incorporate methodologies that allow clinical validation of the reported variables and longitudinal study designs capable of assessing the temporal relationship and potential causal pathways between medication use and the development of depression.

## 5. Conclusions

In this study, we evaluated depressive symptom levels and associated factors among pharmacy students. Consistent with the previous literature, perceived stress and reduced sleep duration were associated with depressive symptoms. Additionally, self-reported medication use showed a modest association with depressive symptomatology; however, the magnitude and clinical significance of this relationship require further investigation. However, given the cross-sectional design and the multifactorial nature of depression, these findings should be interpreted as exploratory observational associations. Future longitudinal studies are needed to clarify the nature of this relationship, assess potential bidirectional pathways, and identify underlying factors that may influence both medication use and depressive symptoms. Overall, these findings contribute to the generation of new research hypotheses regarding medication use and mental health among university students.

## Figures and Tables

**Table 1:** Comparison of risk factors among groups based on PHQ-9 test.

	Students Without Depression or with Mild Symptoms n = 192 (100.0)	Students with Moderate to Severe Depression Symptoms n = 173 (100.0)	*p*-Value
Female gender, n (%)	111 (57.8)	121 (69.9)	0.016
Age, mean ± SD	21.4 ± 1.8	21.8 ± 2.02	0.09
In a relationship, n (%)	89 (46.8)	65 (38.0)	0.09
Lives alone, n (%)	33 (17.2)	26 (15.0)	0.6
Currently work, n (%)	65 (33.9)	76 (43.9)	0.048
Reports any comorbidity, n (%)	13 (6.8)	25 (14.5)	0.016
Physical symptoms, n (%)	100 (52.1)	130 (75.1)	≤0.001
Number of physical symptoms, mean ± SD	0 ± 1	2 ± 1	≤0.001
Social media usage (h/wk), mean ± SD	15.5 ± 10.9	16.3 ± 11.8	0.5
WhatsApp usage, n (%)	96 (50.0)	93 (53.8)	0.4
Instagram usage, n (%)	149 (77.6)	150 (86.1)	0.024
Exercise, n (%)	120 (62.5)	107 (61.8)	0.8
Well rested, n (%)	128 (66.7)	60 (34.7)	<0.001
Pet ownership, n (%)	137 (73.3)	125 (73.1)	0.9
School stress, n (%)	136 (72.0)	151 (88.3)	<0.001
Family problems, n (%)	66 (35.7)	106 (62.0)	<0.001
Psychology help (≥1 yr.), n (%)	14 (7.3)	31 (17.9)	0.002

Abbreviations: SD, standard deviation; h, hours; wks., weeks; yrs: years. Comparisons between proportions were performed using chi-square test and comparisons between means were performed using Student *t*-tests.

**Table 2:** Comparison of risk factors among groups based on PHQ-9 test, excluding students using any psychotropic medications.

	Students Without Depression or with Mild Symptoms n = 185 (100.0)	Students with Moderate to Severe Depression Symptoms n = 159 (100.0)	*p*-Value
Female gender, n (%)	105 (56.8)	112 (70.4)	0.009
Age, mean ± SD	21.5 ± 1.9	21.7 ± 2.02	0.2
In a relationship, n (%)	83 (44.9)	59 (34.1)	0.1
Lives alone, n (%)	31 (16.8)	25 (15.7)	0.7
Currently work, n (%)	65 (35.1)	70 (44.0)	0.09
Reports any comorbidity, n (%)	11 (5.9)	21 (13.2)	0.021
Physical symptoms, n (%)	92 (49.7)	119 (74.8)	<0.001
Number of physical symptoms, mean ± SD	0 ± 1	2 ± 1	<0.001
Social media usage (h/wk), mean ± SD	15.5 ± 11.0	16.2 ± 11.7	0.5
WhatsApp usage, n (%)	92 (49.7)	85 (53.5)	0.4
Instagram usage, n (%)	143 (77.3)	138 (86.8)	0.023
Exercise, n (%)	115 (62.2)	97 (61.0)	0.8
Well rested, n (%)	122 (65.9)	59 (37.1)	<0.001
Pet ownership, n (%)	133 (73.5)	115 (73.2)	0.9
School stress, n (%)	133 (71.9)	142 (89.3)	<0.001
Family problems, n (%)	63 (34.1)	96 (60.4)	<0.001
Psychology help (≥1 yr.), n (%)	11 (5.9)	20 (12.6)	0.032

Abbreviations: SD, standard deviation; h, hours; wks., weeks; yrs: years. Comparisons between proportions were performed using chi-square test and comparisons between means were performed using Student *t*-tests.

**Table 3:** Comparison of pharmacological treatment among groups based on PHQ-9 test.

	Students Without Depression or with Mild Symptoms n = 192 (100.0)	Students with Moderate to Severe Depression Symptoms n = 173 (100.0)	*p*-Value
Medication use (≥2 wks.), n (%)	36 (18.8)	56 (32.4)	0.003
Antidepressants, n (%)	4 (2.1)	14 (8.1)	0.008
Antidiabetic medication, n (%)	1 (0.5)	6 (3.5)	0.040
Antipsychotics, n (%)	1 (0.5)	4 (2.3)	0.1
Anxiolytics, n (%)	3 (1.6)	4 (2.3)	0.6
Antihistamines drugs, n (%)	6 (3.1)	7 (4.0)	0.6
Dermatological drugs, n (%)	4 (2.1)	9 (5.2)	0.1
Hormonal therapy, n (%)	5 (2.6)	12 (6.9)	0.050
Painkillers and NSAIDs, n (%)	9 (4.7)	11 (6.4)	0.4
Psychotropic medications *, n (%)	7 (3.6)	14 (8.1)	0.06
Over-the-counter medications, n (%)	14 (7.3)	19 (11.0)	0.2
Num. of drugs, mean ± SD	0.0 ± 1.0	1.0 ± 1.0	<0.001

Abbreviations: SD, standard deviation. Comparisons between proportions were performed using chi-square test and comparisons between means were performed using Student *t*-tests. * Composed of patients using antidepressants, antipsychotics or anxiolytics.

**Table 4:** Comparison of self-reported physical symptoms.

	Students Without Depression or with Mild Symptoms n = 192 (100.0)	Students with Moderate to Severe Depression Symptoms n = 173 (100.0)	*p*-Value
Previous diagnosis of depression *, n (%)	10 (5.2)	41 (23.7)	<0.001
Vision problems, n (%)	28 (14.6)	46 (26.6)	0.004
Recurrent headaches, n (%)	43 (22.4)	78 (45.1)	<0.001
Gastrointestinal symptoms, n (%)	26 (13.5)	50 (28.9)	<0.001
Musculoskeletal symptoms, n (%)	26 (13.5)	46 (26.6)	0.002
Respiratory symptoms, n (%)	4 (2.1)	13 (7.5)	0.014
Skin conditions, n (%)	13 (6.8)	28 (16.2)	0.004
Hormonal disorders, n (%)	11 (5.7)	39 (22.5)	<0.001
Recurrent infections, n (%)	5 (2.6)	8 (4.6)	0.2

Comparisons between proportions were performed using the chi-square test. * Students self-reported whether they had ever received a diagnosis of depression from a healthcare professional.

**Table 5:** Factors associated with PHQ-9 scores in the multivariable general linear model.

Variable	F	*p*-Value	Partial η^2^
Gender	1.091	0.2	0.003
Age	2.299	0.1	0.007
In a relationship	1.286	0.2	0.004
Currently working	2.401	0.1	0.007
Comorbidity	3.304	0.07	0.009
Insufficient sleep	50.835	<0.001	0.128
School stress	18.716	<0.001	0.051
Psychological help (≥1 year)	6.947	0.009	0.020
Social media use (hours)	0.259	0.6	0.001
Number of drugs	4.389	0.037	0.012

F tests derive from the general linear model; *p* values reported as in the original table (values of 0.000 shown as <0.001). Partial η^2^ quantifies effect size.

**Table 6:** Factors associated with PHQ-9 scores in the multivariable logistic regression model.

	Moderate to Severe Depressive Symptoms					
Variable	OR	95% CI	*p*	aOR	95% CI	*p*
Gender	0.70	0.42–1.15	0.1	---	---	---
Age	1.08	0.95–1.23	0.2	---	---	---
In a relationship	0.57	0.35–0.91	0.021	0.61	0.38–0.98	0.042
Currently working	1.69	1.03–2.78	0.037	1.73	1.07–2.77	0.023
Comorbidity	1.62	0.71–3.70	0.2	---	---	---
Insufficient sleep	3.12	1.96–4.97	<0.001	3.37	2.13–5.31	<0.001
School stress	2.88	1.51–5.49	0.001	2.80	1.50–5.22	0.001
Psychological help (≥1 year)	0.48	0.22–1.03	0.06	0.45	0.21–0.96	0.039
Social media use (hours)	1.01	0.98–1.02	0.7	---	---	---
Number of drugs	1.30	0.94–1.78	0.1	1.42	1.05–1.92	0.022

Abbreviatures: OR, odds ratios; CI, confidence intervals; aOR, adjusted odds ratios. Crude ORs were obtained using the enter method. aOR was obtained using the stepwise method.

## Data Availability

The data presented in this study are available from the corresponding author upon reasonable request. Because the dataset contains information from a vulnerable population, access to the data is subject to review and approval to ensure the protection of participant privacy, confidentiality, and compliance with ethical requirements.

## Abbreviations

The following abbreviations are used in this manuscript:

NSAIDs	Nonsteroidal anti-inflammatory drugs
PHQ-9	Patient Health Questionnaire-9
SD	Standard Deviation
SNRIs	Serotonin–norepinephrine reuptake inhibitors
SSRIs	Selective serotonin reuptake inhibitors
Wks.	Weeks
Yr.	Year
